# IL-22 increases the production of sFRP3 by FLS in inflammatory joint diseases

**DOI:** 10.1590/1414-431X20209880

**Published:** 2020-08-03

**Authors:** G.G. Resende, C.R.L. Machado, M.A. Rocha, R.B.V. Macedo, J.S.S. Bueno, A.M. Kakehasi, M.V. Andrade

**Affiliations:** 1 Serviço de Reumatologia, Hospital das Clínicas, Universidade Federal de Minas Gerais, Belo Horizonte, MG, Brasil; 2 Departamento de Clínica Médica, Faculdade de Medicina, Universidade Federal de Minas Gerais, Belo Horizonte, MG, Brasil; 3 Departamento de Estatística, Universidade Federal de Lavras, Lavras, MG, Brasil; 4 Programa de Pós Graduação em Ciências Aplicadas à Saúde do Adulto, Faculdade de Medicina, Universidade Federal de Minas Gerais, Belo Horizonte, MG, Brasil

**Keywords:** L-22, Wnt signaling pathway, Fibroblast-like synoviocytes, Rheumatoid arthritis, Spondyloarthritis

## Abstract

Rheumatoid arthritis (RA), psoriatic arthritis (PsA), and ankylosing spondylitis (AS) are inflammatory diseases with different bone remodeling patterns. Fibroblast-like synoviocytes (FLS) are cells involved in the transition from an acute and reparable phase to a chronic and persistent stage in these diseases. The distinction of joint phenotypes involves inflammatory cytokines such as tumor necrosis factor alpha (TNF-α), interleukin (IL)-17, and IL-22 directly or through key signaling pathways such as Wnt. To evaluate the role of FLS as the source of Wnt antagonists (sFRP3/FRZB and Dkk1) in the synovia, levels of TNF- α, IL-17, IL-22, Dkk1, and sFRP3 were measured by ELISA directly in the synovial fluid of patients with RA, PsA, or AS. Dkk1 and sFRP3 were also measured in the FLS culture supernatants after different inflammatory stimulus. sFRP3 and Dkk1 are constitutively expressed by FLS. IL-22 and sFRP3 were positively correlated (r=0.76; P<0.01) in synovial fluid. The stimulation of FLS with IL-22, but not TNF-alpha and IL-17, increased the production of sFRP3. No stimulus altered the basal expression of Dkk1. These results showed, for the first time, the ability of IL-22 to increase the expression of sFRP3/FRZB by human FLS in both *in vitro* and *ex vivo* models. This finding linked IL-22 to local inhibition of Wnt signaling and possibly to blockade of osteogenesis. Furthermore, FLS presented as a source of this inhibitor in synovial fluid, assigning to this cell a bone injury mechanism.

## Introduction

Rheumatoid arthritis (RA), psoriatic arthritis (PsA), and ankylosing spondylitis (AS) are inflammatory joint diseases (IJD) that exhibit distinct bone remodeling patterns. Bone erosions are the most typical feature seen in RA while new bone formation is the mark of AS. PsA, interestingly, presents as a combination of these patterns with bone formation and erosions being observed simultaneously in the same patient and even in the same joint (1).

Different biological systems regulate bone remodeling and should be involved in the distinctiveness of tissue damage in each disease. Inflammatory cytokines such as tumor necrosis factor alpha (TNF-α) and interleukins 17 (IL-17) and 22 (IL-22) act modulating both osteoclast- and osteoblastogenesis, directly or indirectly through other systems as the axis composed by the receptor activator of nuclear factor kappa-B (RANK), its ligand (RANKL), and its decoy receptor osteoprotegerin (OPG), and signaling pathways such as Hedgehog (Hh), bone morphogenetic proteins (BMP), and Wingless/Integrated (Wnt).

The Wnt signaling pathway is a highly conserved group of signal transduction pathways involved in bone formation and bone repair from embryogenesis to adult life. It encompasses the soluble Wnt ligands (19 currently described in humans) and its extracellular inhibitors, such as Dickkopf-related protein 1 (Dkk1), secreted Frizzled-related proteins (sFRP), and sclerostin (SOST). Collectively, and often interacting, the effects of these systems impact on the differentiation of joint phenotypes (2–5).

The fibroblast-like synoviocytes (FLS), a cell type decisively involved in the pathophysiological processes of synovitis in many joint diseases, are currently considered a component of innate immunity. FLS expresses pathogen recognition receptors (PRRs), secretes inflammatory cytokines such as IL-6, chemokines such CCL2 (previously monocyte chemoattractant protein-1 - MCP1) in addition to metalloproteinases (MMPs) and RANKL. FLS is also responsible for the perpetuation of local inflammation involved in the transition from an acute and repairable phase to a chronic and irreversible stage in chronic arthritis (6).

This study evaluated the role of FLS as a source of Wnt antagonists (sFRP3/FRZB and Dkk1) in the synovial environment and the contribution of inflammatory cytokines (IL-17, IL-22, and TNF-α) to this function.

## Material and Methods

### Patients

Twenty-one patients were consecutively selected for arthrocentesis. Three PsA patients as defined by the CASPAR criteria of 2006 (7), 15 RA patients according to the ACR criteria of 1990 (8), and thee AS patients based on the New York modified criteria of 1984 (9). All patients had disease activity measured as moderate to high according to the appropriate index for each diagnosis (DAS28-CRP for RA and PsA; ASDAS-CRP for AS) and were indicated by their attending physician for articular infiltration in at least one large joint, regardless of their systemic treatment.

### FLS culture

Briefly, after synovial fluid (SF) centrifugation (300 *g*; 15 min; 37°C), the pellet was seeded onto culture plates containing Dulbecco's modified Eagle medium (DMEM) (Gibco, USA, ref.: 12800-017) supplemented with 15% fetal bovine serum (FBS) (Sigma, USA ref.: M-0643), 1% non-essential amino acids (Gibco, ref.: 11140-050), 10,000 U/10,000 U penicillin-streptomycin (Gibco, ref.: 15140-122), and 1% amphotericin B (Gibco, ref.: 15245-012). The cultures were kept at 37°C and 5% CO_2_, with medium exchange twice a week until reaching enough cell quantity, when cultures were stored in aliquots at -80°C. We limited the use to the fourth to eighth passages in all experiments since it already has been demonstrated that in this interval, FLS represents the predominant cell type (>95% positive markers for fibroblasts and negative for macrophages) while minimizing the effect of cell senescence (10–13).

### Enzyme-linked immunosorbent assays (ELISA)

Dkk1, sFRP3/FRZB, and IL-22 concentrations were measured directly in the synovial fluid (n=19; 13 RA, 3 PsA, and 3 AS) and in FLS culture supernatants, following the manufacturer's protocols (R&D systems, ref.: DY1906; DY192; DY210; DY317; and DY782). A pilot assay (n=3) for defining the optimal dose and time-to-collect was performed using one sample source from each IJD, none of them using synthetic or biological disease-modifying antirheumatic drugs, but only using non-steroidal anti-inflammatory drugs (NSAIDs) and corticosteroids (2.5-10 mg of prednisone/day). The FLS culture supernatant was collected at 24, 48, and 72 h after stimulus with recombinant human TNF-α (Peprotech, USA, ref.: 300-01A) or recombinant human IL-17A (Peprotech, ref.: 200-17), both at concentrations of 1, 10, 50, or 100 ng/mL, or recombinant human IL-22 (Peprotech, ref.: 200-22) at doses of 1, 10, 100, or 200 ng/mL. There were controls (both cells supernatants without stimulation and culture medium without cells) for all samples. Based on the results of this pilot study, the concentrations of sFRP3 were measured in a larger sample (n=11: 5 RA, 3 PsA, and 3 AS), 24 h after stimulus with IL-22, TNF-α, or IL-17, all at 10 ng/mL. All experiments were performed in three complete and independent repetitions.

### Statistical analysis

Fisher's exact test was used to compare frequencies, Spearman's test to evaluate the correlation between continuous variables, the randomization test and the Wilcoxon-Mann-Whitney U test for non-normally distributed variables, and an ANOVA F test and Student's *t*-test for normally distributed variables to compare results from different stimuli. The R program version 3.2.3 (<r-project.org>) and GraphPad Prism program (USA) version 6.01 were used for statistical analyses. P values <0.05 were considered to be statistically significant.

## Results

The demographic and clinical data of patients are shown in Table 1. There were no statistically significant differences of gender, age, duration of symptoms, acute inflammatory markers, and use of synthetic or biological immunosuppressant and steroids among the three diagnostic groups. A statistically significant difference was observed only for the use of NSAIDs, which was more common in PsA and AS (P<0.02), and the positivity of the rheumatoid factor, which was more frequent in RA (P<0.01).

**Table 1:** Demographic and clinical data of patients with rheumatoid arthritis (RA), psoriatic arthritis (PsA), and ankylosing spondylitis (AS).

	RA	PsA	AS	All
Sample size (n)	15	3	3	21
Gender (% women)	73.3	66.6	33.3	66.6
Age (years)	53.2 (12.2)	53.5 (12.9)	42.4 (12.1)	51.4 (11.4)
Duration of symptoms (years)	13.7 (13.8)	3.3 (3.2)	15.8 (13.2)	12.5 (9.6)
Positive rheumatoid factor (%)	86.6	0	0	61.9
ESR (mm/1^st^ hour)	30.1 (26.4)	28.0 (20.8)	64.2 (37.8)	34.5 (27.8)
CRP (mg/L)	27.6 (22.0)	22.6 (17.8)	49.2 (33.3)	29.8 (19.8)
Glucocorticoid use (%)	53.3	33.3	33.3	47.6
Prednisone daily doses (mg)	8.1 (3.5)	10 (1.5)	3.8 (1.8)	7.8 (2.9)
NSAID use (%)	33.3	66.6	100	47.6
Synthetic DMARD use (%)	80.0	66.6	33.3	71.4
Biological DMARD use (%)	13.3	33.3	33.3	23.8

Data are reported as means±SD or percentage. ESR: erythrocyte sedimentation rate; CPR: C-reactive protein; NSAID: non-steroidal anti-inflammatory drugs; DMARD: disease-modifying antirheumatic drugs.

**Figure 1:**
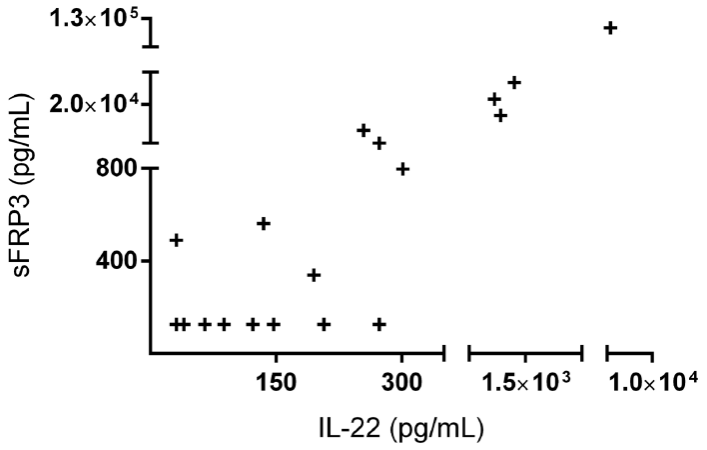
Correlation between interleukin (IL)-22 and secreted Frizzled-related protein (sFRP)3 synovial concentrations. IL-22 and SFRP3/FRZB concentrations measured in synovial fluid of 19 patients with different inflammatory joint diseases (two of these presented the exact same pair value so only 18 dots are shown). The nine lower values of sFRP3 and the three lower values of IL-22 represent imputed data of observations below the method’s detection limit (informed by the manufacturer as being 31.1 pg/mL for IL-22 and 125 pg/mL for sFRP3). Spearman r coefficient=0.76. P=0.002.

There were no statistically significant differences in the synovial fluid concentrations of IL-17, IL-22, TNF-α, sFRP3/FRZB, and Dkk1, duration of symptoms, and use of immunosuppressants, glucocorticoids, or NSAIDs among different diseases. IL-22 and sFRP3/FRZB concentrations in the synovial fluid were positively correlated with a Spearman coefficient of 0.76 and P<0.01, although 16% (3/19) and 47% (9/19) of samples presented censored values of IL-22 and sFRP3, respectively, lower than the method’s detection limits (Figure 1).

**Figure 2:**
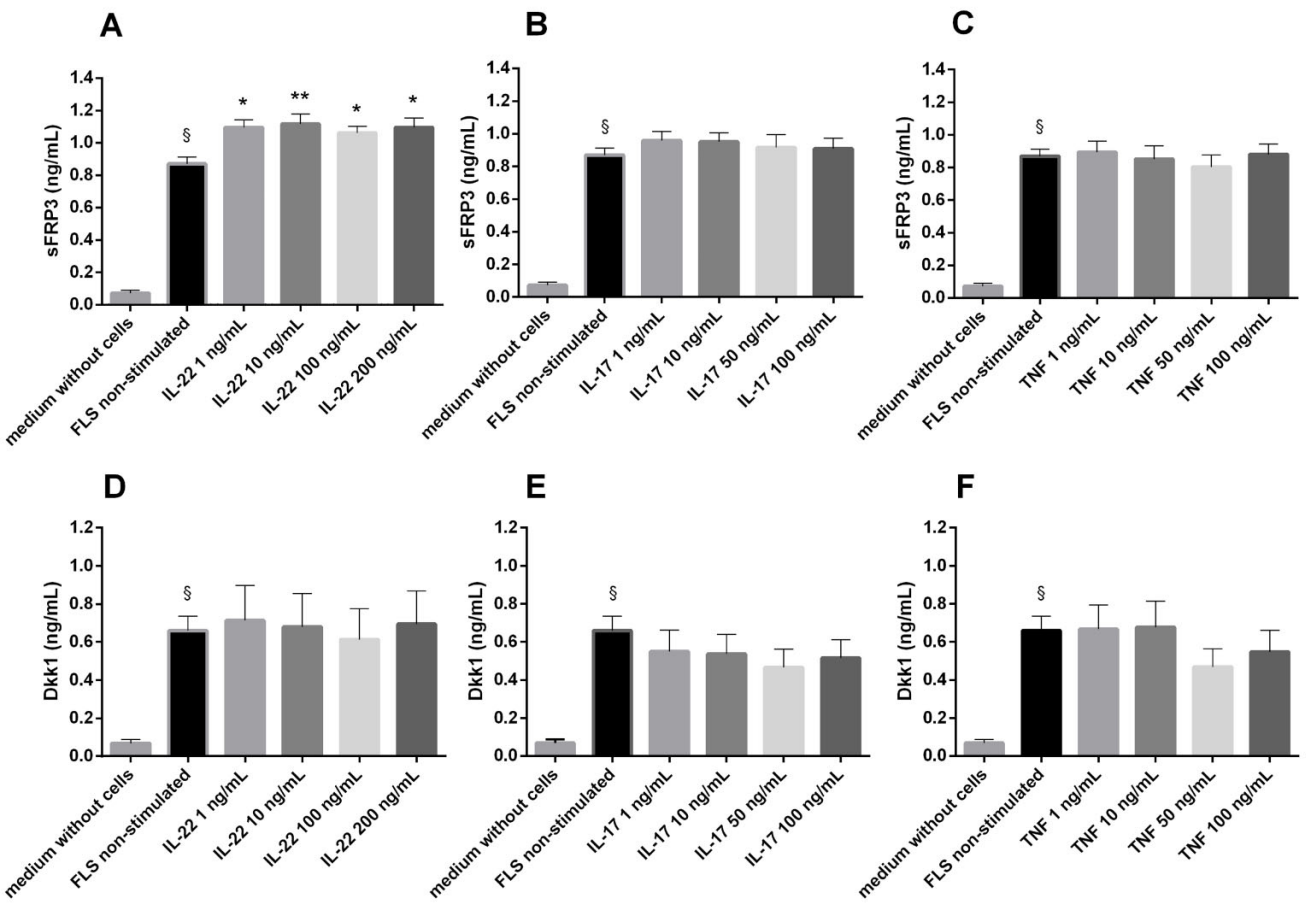
Wnt inhibitor expression by fibroblast-like synoviocytes (FLS) after different cytokine stimuli. Secreted Frizzled-related proteins (sFRP3)/FRZB (**A-C**) and Dickkopf-related protein 1 (Dkk1) (**D-F**) concentrations measured in the FLS culture supernatant (n=3) after different stimuli (interleukin (IL)-17, IL-22, and tumor necrosis factor (TNF)-α) and in controls. Data are reported as mean(±SE) from three independent repetitions. ^§^P<0.0001 (*vs* medium without cells). *P<0.05 and **P<0.01 (stimulated *vs* non-stimulated FLS) (ANOVA).

sFRP3/FRZB and Dkk1 were constitutively expressed by FLS in all diseases, as could be observed in the pilot experiment (n=3) by the presence of these proteins even in the unstimulated FLS supernatant, in contrast with the negative controls (medium without cells). Stimulation with IL-22 increased the expression of sFRP3/FRZB by FLS mainly when the supernatant was collected 24 h after stimulus with 10 ng/mL. For Dkk1 concentration, no difference was observed when different stimuli (IL-17, IL-22, or TNF-α), at the doses used, were compared (Figure 2). There was no significant difference between different collection times after the same stimulus or between different diagnoses.

**Figure 3:**
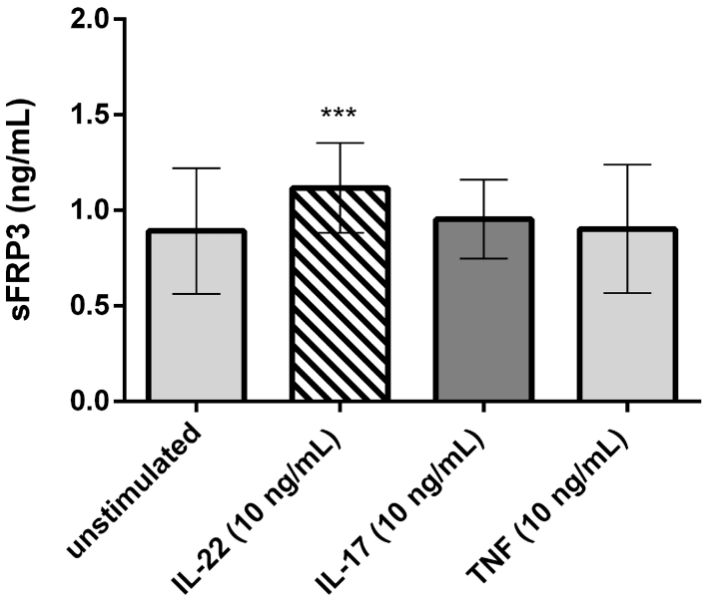
Secreted Frizzled-related proteins (sFRP3) in the supernatant of fibroblast-like synoviocytes (FLS) culture supernatant (n=11) treated with interleukin (IL)-22, IL-17, or tumor necrosis factor (TNF-α), all of them at a dose of 10 ng/mlL, and in untreated controls. Data are reported as the means±SD from three independent repetitions. ***P<0.001 *vs* unstimulated FLS (ANOVA).

The larger assay (n=11) confirmed that IL-22, but not IL-17 or TNF-α, was capable of inducing an increase in the expression of sFRP3/FRZB by FLS. The mean (SE) of concentrations was 0.89±0.03 ng/mL for unstimulated and 1.12±0.04 ng/mL for post-IL-22 (P=0.0003), as exhibited in Figure 3. This finding was similar even when the three different IJD were separately analyzed (data not shown).

## Discussion

Our results showed, for the first time, the capacity of IL-22 to increase the expression of sFRP3/FRZB by FLS. This result agreed with the high correlation observed between the concentrations of these two proteins when directly measured in the synovial fluid. These findings may attribute another role to IL-22 as a local inhibitor of Wnt signaling and therefore osteogenesis blockade. Furthermore, FLS appeared to be a source of this inhibitor in synovial fluid, assigning to this cell another bone injury mechanism. Kim et al. showed a similar relationship between IL-22 and RANKL, which also contributes to inflammation-associated bone loss (14). Similarly, experimental models have demonstrated the role of IL-22 in bone resorption (15,16). In contrast, a study by Sherlock et al. (17) suggested that IL-22 mediates the osteoproliferative component of spondyloarthritis (SpA), inducing the entheseal expression of osteogenesis genes. The discrepancy between these results may be due to the functional ambiguity of IL-22, as it has been shown to perform opposing actions when operating in a different cellular milieu and when exposed to different costimulatory profiles (16). Ijiri et al. (18) demonstrated sFRP3 expression in synoviocytes, but this was observed predominantly in macrophage-like (type A) and not in FLS (type B). Furthermore, their evaluation was limited to the transcription level (mRNA), with no protein measurement, as performed in our study.

No stimuli in this present study increased the expression of Dkk1. There are conflicting conclusions about this issue in the literature. Some authors demonstrated that TNF-α induces Dkk1 expression by synoviocytes (19,20), including mouse and human FLS. Contrariwise, Hardy et al. (21) showed that glucocorticoids directly regulated the expression of Dkk1 by FLS but not by inflammatory cytokines and Lavocat et al. (22) reported a decrease in Dkk1 expression induced by treatment with TNF-α and/or IL-17. Taken together, these data suggest that this topic is still controversial.

A limitation of our study is the small number of PsA and AS samples that did not allow possible differences to be detected in protein expressions by FLS from different diseases. Besides this, the presence of lower values of IL-22 and sFRP3 in some samples of synovial fluid may be due to individual differences, but also to a worse assay performance in the synovial fluid owing to its viscosity. Despite this limitation, we can recognize it as a pilot study, and it was able to demonstrate that FLS responded to IL-22 regardless of donor joint disease.

We concluded that IL-22 increased the expression of sFRP3/FRZB by FLS in both *in vitro* and *ex vivo* models, which could mean that IL-22 is a local Wnt inhibitor. FLS from patients with three IJD with different pathogenetic mechanisms (RA, PsA, and AS) constitutively expressed Wnt inhibitors (sFRP3 and Dkk1) setting this cell as a bone injury element in joint pathology. Further studies, with larger samples, are needed to confirm these data and investigate differences between diseases with distinct tissue remodeling patterns.
